# Usefulness of a questionnaire for assessing the relationship between eating behavior and steatotic liver disease among Japanese male young adults

**DOI:** 10.1038/s41598-024-52797-8

**Published:** 2024-01-25

**Authors:** Takao Miwa, Satoko Tajirika, Tatsunori Hanai, Nanako Imamura, Miho Adachi, Ryo Horita, Taku Fukao, Masahito Shimizu, Mayumi Yamamoto

**Affiliations:** 1 Health Administration Center, Gifu University, 1-1 Yanagido, Gifu, 501-1193 Japan; 2 Department of Gastroenterology/Internal Medicine, Graduate School of Medicine, Gifu University, Gifu, Japan; 3 Gifu University Hospital, Gifu, Japan; 4 United Graduate School of Drug Discovery and Medical Information Sciences, Gifu University, Gifu, Japan

**Keywords:** Hepatology, Obesity

## Abstract

This study aimed to reveal the relationship between eating behavior and nonalcoholic fatty liver disease (NAFLD)/metabolic dysfunction-associated steatotic liver disease (MASLD) in young adults and suggest a questionnaire for eating behavior assessment. We included 322 male graduate students at Gifu University. Diagnoses of NAFLD and MASLD were based on the presence of hepatic steatosis on ultrasonography. Eating behavior was assessed using the eating behavior questionnaire (EBQ) recommended by the Japan Society for the Study of Obesity. We assessed the eating behaviors associated with NAFLD and MASLD using logistic regression, decision tree, and random forest analyses. The median age of the participants was 22 years, and 16% and 11% had NAFLD and MASLD, respectively. The EBQ total score was significantly higher in participants with MASLD than in those without MASLD (102 vs. 90 points, *P* = 0.006) and in those with NAFLD than in those without NAFLD (97 vs. 90 points, *P* = 0.007). Among eating behavior categories, the decision tree and random forest analyses revealed that “perception of constitution and weight” was the strongest contributor for NAFLD/MASLD. Our study revealed that eating behavior assessed with the EBQ is robustly associated with NAFLD and MASLD in young male adults.

## Introduction

Owing to the increasing prevalence of obesity, type 2 diabetes, and metabolic syndrome, the causes and burden of liver cirrhosis are dramatically changing^1^. Viral hepatitis remains an important cause of cirrhosis. However, increasing vaccination coverage and effective antivirals have contributed to improved death rates due to hepatitis B-associated cirrhosis^2^. Furthermore, effective, directly acting antivirals have revolutionized the treatment of hepatitis C, and the burden in patients with hepatitis C is forecasted to improve^3^. In contrast, nonalcoholic fatty liver disease (NAFLD) and nonalcoholic steatohepatitis are rapidly growing etiologies of liver cirrhosis, which lead to decompensation, liver cancer, and mortality^4^. Recently, a panel of international experts proposed metabolic dysfunction-associated steatotic liver disease (MASLD) as a new term for fatty liver disease^5,6^. The definition of MASLD is based on the risk profiles of NAFLD; accordingly, MASLD is expected to better identify patients at high risk for disease burden of chronic liver disease than NAFLD. Therefore, more efforts are required for the early prevention, detection, and intervention of NAFLD and MASLD to improve outcomes.

Lifestyle modifications consisting of a healthy diet and regular exercise form the foundation for the treatment of patients with NAFLD and MASLD^7–10^. However, it is difficult to achieve lifestyle modification in patients with NAFLD because some patients are not in the active phase of lifestyle modification^11^. Behavioral therapy is an important component of lifestyle modification based on learning theory, which assumes that behaviors that result in obesity have a strong educational component and, therefore, can be modified or relearned^12^. Given its effect on treatment outcomes, major guidelines recommend behavioral interventions, including motivational interviewing, stimulus control, cognitive restriction, and self-monitoring, for the treatment of obesity^13^. The Japan Society for the Study of Obesity suggested an eating behavior questionnaire (EBQ) as an initial step in assessing eating behavior and providing effective behavioral therapy for patients with obesity^14^. However, very few attempts have been made to assess eating behavior and behavioral therapy in patients with NAFLD/MASLD.

The primary aim of this study was to evaluate the association between eating behavior and NAFLD/MASLD among Japanese male young adults. The secondary aim was to develop a useful EBQ to stratify the risk of NAFLD/MASLD among young Japanese male adults.

## Methods

### Study design and participants

This cross-sectional study included 322 male graduate students who enrolled at Gifu University (Gifu, Japan) and underwent health checkups for new students in April 2022 (Supplementary Fig. S1). The exclusion criteria included severe comorbidities and international students. Given the limit for alcohol consumption in the diagnosis of NAFLD/MASLD, participants with an alcohol intake > 30 g/day were also excluded from the study^5–9^. The study aims were explained to the candidates and written informed consent was obtained from all participants. The study protocol was reviewed and approved by the Institutional Review Board of the Graduate Medical School, Gifu University (approval no. 2021-B167), and conformed to the provisions of the Declaration of Helsinki (as revised in Fortaleza, Brazil, October 2013).

### Clinical and biochemical characteristics

Clinical and biochemical characteristics were assessed using health checkup data. Mild alcohol intake was defined as an alcohol intake permitted within the diagnosis of NAFLD/MASLD (≤ 30 g/day)^5–9^. Laboratory data assessed included aspartate aminotransferase (AST), alanine aminotransferase (ALT), triglycerides, high-density lipoprotein (HDL) cholesterol, low-density lipoprotein cholesterol, hemoglobin A1c (HbA1c), and fasting or casual glucose levels.

### Diagnosis of NAFLD and MASLD

Hepatic steatosis was evaluated using liver ultrasound (CX50; Koninklijke Philips N.V., Amsterdam, Netherlands) performed by a single hepatologist blinded to the laboratory data. Participants with hepatic steatosis without other liver diseases, such as alcohol-related liver disease (alcohol intake > 30 g/day), viral liver disease, or drug-induced liver disease, were diagnosed with NAFLD^8,9^. MASLD was diagnosed in participants with hepatic steatosis who met at least one of the following cardiometabolic criteria: overweight (body mass index [BMI] ≥ 23 kg/m^2^ or waist circumference > 94 cm); impaired glucose tolerance (fasting blood glucose ≥ 100 mg/dL, occasional blood glucose ≥ 140 mg/dL, HbA1c ≥ 5.7%, type 2 diabetes mellitus, or treatment with antidiabetic medicine); hypertension (blood pressure ≥ 130/85 mmHg); hypertriglyceridemia (triglyceride level ≥ 150 mg/dL); low HDL cholesterol (< 40 mg/dL); or received any treatment related to these conditions^5,6^.

### Assessment of eating behavior questionnaire

An EBQ recommended by the Japan Society for the Study of Obesity was administered at the time of health checkups^14^. The questionnaire consisted of 55 items and participants were asked to answer each item with the following scales: “not at all like me” (one point), “sometimes like me” (two points), “tendency like me” (three points), and “extremely like me” (four points) (Supplementary Table S1). Eating behavior was assessed by summarizing the score of each item into seven categories: (1) perception of constitution and weight, (2) motivation for eating, (3) eating as diversion, (4) feeling of fullness and hunger, (5) bad eating habits, (6) contents of meals, and (7) unsteady eating pattern. The subtotal scores of all categories were summarized as total scores (Supplementary Table S2). A higher score in each category and total score indicated worse eating behavior. An octagonal diagram was created using the score for each category as a percentage^14^.

### Objectives

The primary objective of this study was to examine the association between the EBQ total score and presence of NAFLD/MASLD to reveal the relationship between eating behavior and NAFLD/MASLD among Japanese male young adults. The secondary objective was to explore the association between each EBQ category and the presence of NAFLD/MASLD to clarify the characteristics of eating behavior associated with NAFLD/MASLD among young Japanese male adults.

### Statistical analyses

Continuous variables are expressed as medians and interquartile ranges. Categorical variables are expressed as numbers and percentages (%). Groups were compared using the Mann–Whitney *U* test or chi-square test. The association between eating behavior and NAFLD/MASLD was assessed using logistic regression analysis. The results are presented as odds ratios (OR) and 95% confidence intervals (CI). The discriminative ability of the EBQ to identify NAFLD/MASLD was assessed using receiver operating characteristic (ROC) curve analysis, shown as the area under the curve (AUC), and the optimal cutoff value was identified using the Youden’s index. A restricted cubic spline (RCS) model with three knots was evaluated to demonstrate the association between the EBQ scores and NAFLD/MASLD^15^. The reference point of the EBQ total score was manually adjusted to 90 points considering the median value of the EBQ total score, and the bin width was set to two points. Multivariate analysis, including all categories of the EBQ, was performed to determine the strongest eating behavior category for MASLD and NAFLD. The decision tree analysis was performed to identify eating behavior that classifies MASLD and NAFLD^16^. Random forest analysis was performed to identify eating behaviors that contributed to MASLD and NAFLD, expressed as variable importance values^16^. All tests were two-sided, and *P* < 0.05 was set as the threshold for statistical significance. All statistical analyses were performed using JMP (version 16.2.0, SAS Institute Inc., Cary, NC, USA) and R (version 4.3.1, R Foundation for Statistical Computing, Vienna, Austria).

## Results

### Characteristics of study participants

The participants’ characteristics are listed in Table 1. Of the 322 participants, the median age was 22 years, and 8%, 36%, and 40% reported current or former smoking habits, mild alcohol consumption, and exercise habits, respectively. The median waist circumference and BMI were 76 cm and 20.7 kg/m^2^, respectively. Regarding the cardiometabolic criteria, the prevalence of overweight (BMI ≥ 23 kg/m^2^), impaired glucose tolerance, hypertension, hypertriglyceridemia, and low HDL cholesterol were 22%, 2%, 24%, 16%, and 4%, respectively. Of the 322 participants, 17% (n = 54) were diagnosed with hepatic steatosis by ultrasound. MASLD and NAFLD were diagnosed in 11% (n = 36) and 16% (n = 54) of participants, respectively; of these, 11% (n = 36) had both MASLD and NAFLD (Supplementary Fig. S2).

**Table 1:** Characteristics of Japanese male young adults divided by MASLD or NAFLD.

Characteristic	All participants (n = 322)	MASLD (n = 36)	Non-MASLD (n = 286)	*P-*value	NAFLD (n = 54)	Non-NAFLD (n = 268)	*P-*value
Demographics							
Age (years)	22 (22–23)	22 (22–23)	22 (22–22)	0.026	22 (22–23)	22 (22–22)	0.046
Current/former smoking, n (%)	25 (8)	2 (6)	23 (8)	0.599	4 (7)	21 (8)	0.945
Mild alcohol intake, n (%)	116 (36)	14 (42)	102 (35)	0.419	22 (41)	94 (35)	0.429
Exercise habits, n (%)	128 (40)	13 (36)	115 (40)	0.636	21 (39)	107 (40)	0.887
Physical examination							
Waist circumference (cm)	76 (72–81)	85 (79–101)	75 (72–80)	< 0.001	82 (77–92)	75 (72–79)	< 0.001
Body mass index (kg/m^2^)	20.7 (19.3–22.7)	25.0 (22.8–28.6)	20.3 (19.2–22.1)	< 0.001	23.4 (22.7–26.8)	20.2 (19.0–22.0)	< 0.001
Cardiometabolic criteria							
Overweight, n (%)	72 (22)	26 (72)	46 (16)	< 0.001	30 (56)	42 (16)	< 0.001
Impaired glucose tolerance, n (%)	6 (2)	2 (6)	4 (1)	0.082	2 (4)	4 (1)	0.273
Hypertension, n (%)	76 (24)	27 (75)	49 (17)	< 0.001	27 (50)	49 (18)	< 0.001
Hypertriglyceridemia, n (%)	50 (16)	20 (56)	30 (10)	< 0.001	20 (37)	30 (11)	< 0.001
Low HDL cholesterol, n (%)	13 (4)	6 (17)	7 (2)	< 0.001	6 (11)	7 (3)	0.004
Laboratory test							
AST (IU/L)	18 (15–22)	22 (17–30)	18 (15–21)	< 0.001	21 (17–27)	18 (15–21)	< 0.001
ALT (IU/L)	20 (16–27)	36 (25–57)	19 (16–25)	< 0.001	30 (23–49)	19 (15–25)	< 0.001
Triglycerides (mg/dL)	79 (56–117)	157 (89–228)	73 (55–103)	< 0.001	128 (69–176)	74 (55–101)	< 0.001
HDL cholesterol (mg/dL)	57 (49–64)	49 (43–58)	58 (50–64)	< 0.001	54 (45–61)	57 (50–64)	0.608
LDL cholesterol (mg/dL)	96 (81–111)	97 (83–120)	96 (81–110)	0.512	99 (83–117)	95 (81–110)	0.561
HbA1c (%)	5.2 (5.1–5.3)	5.3 (5.2–5.5)	5.2 (5.0–5.3)	< 0.001	5.3 (5.2–5.4)	5.2 (5.0–5.3)	< 0.001

Values are presented as number (percentage) or median (interquartile range). Analysis was performed using the chi-square test or Mann–Whitney *U* test.

*ALT* alanine amino transferase, *AST* aspartate aminotransferase, *HbA1c* hemoglobin A1c, *HDL* high-density lipoprotein, *LDL* low-density lipoprotein, *MASLD* metabolic dysfunction-associated steatotic liver disease, *NAFLD* nonalcoholic fatty liver disease.

### Impact of eating behavior on MASLD and NAFLD in Japanese male young adults

**Figure 1:**
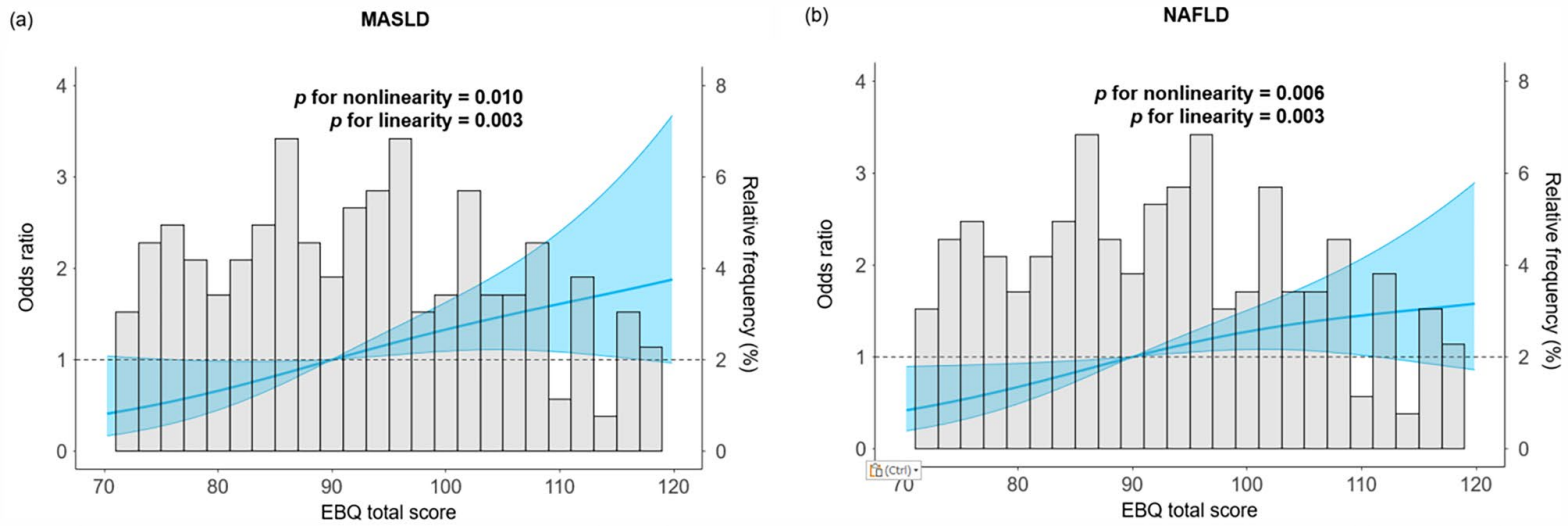
Age-adjusted odds ratio for the association between (**a**) MASLD, (**b**) NAFLD, and EBQ total score. The analyses were performed using the multivariate logistic regression models with restricted cubic spline with three knots and the reference points were manually adjusted. *EBQ* eating behavior questionnaire, *MASLD* metabolic dysfunction-associated steatotic liver disease, *NAFLD* nonalcoholic fatty liver disease.

**Figure 2:**
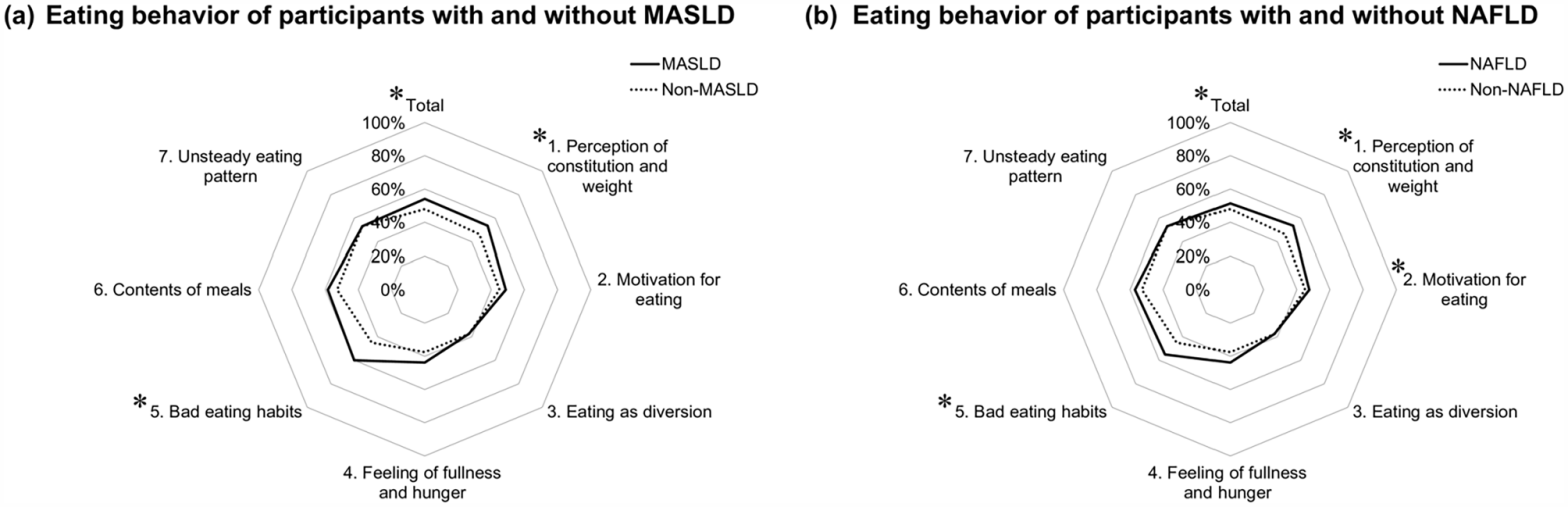
Comparison of octagon diagram plotting eating behavior in participants with and without (**a**) MASLD and (**b**) NAFLD. *Statistically significant (*P* < 0.05) in the Mann–Whitney *U* test. *MASLD* metabolic dysfunction-associated steatotic liver disease, *NAFLD* nonalcoholic fatty liver disease.

A comparison of eating behavior scores between participants with and without MASLD or NAFLD is shown in Table 2. Participants with MASLD had significantly higher EBQ total scores than those without MASLD (102 vs. 90 points; *P* = 0.009). The RCS models demonstrated that the risk of MASLD (Fig. 1a) and NAFLD (Fig. 1b) increased as the EBQ total score elevated. The ROC curve analysis identified a EBQ total score > 100 points as an optimal cutoff value to identify MASLD (AUC, 0.63), with sensitivity and specificity of 0.53 and 0.70, respectively. In the same manner, the ROC curve analysis identified a EBQ total score > 82 points as an optimal cutoff value to identify NAFLD (AUC, 0.62), with sensitivity and specificity of 0.83 and 0.34, respectively. In each category, participants with MASLD had significantly higher scores for “perception of constitution and weight” (15 vs. 13 points; *P* < 0.001) and “bad eating habits” (12 vs. 9 points; *P* = 0.002) compared to those without MASLD (Fig. 2a). Similarly, participants with NAFLD had significantly higher EBQ total scores than those without NAFLD (97 vs. 90 points; *P* = 0.007). In each category, participants with NAFLD had significantly higher scores in “perception of constitution and weight” (15 vs. 13 points; *P* < 0.001), “motivation for eating” (19 vs. 18 points; *P* = 0.044), and “bad eating habits” (11 vs. 9 points; *P* = 0.006) compared to those without NAFLD (Fig. 2b). Subgroup analyses revealed that participants with both NAFLD and MASLD had significantly higher EBQ total scores than those without NAFLD/MASLD (102 vs. 90 points; *P* = 0.022), while there was no statistical difference between participants with only NAFLD and those without NAFLD/MASLD (92 vs. 90 points; *P* = 0.915) (Supplementary Fig. S3).

**Table 2:** Scores of eating behavior questionnaire in Japanese male young adults with and without MASLD or NAFLD.

Category	MASLD (n = 36)	Non-MASLD (n = 286)	*P-*value	NAFLD (n = 54)	Non-NAFLD (n = 268)	*P-*value
Perception of constitution and weight	15 (13–19)	13 (10–15)	< 0.001	15 (13–18)	13 (10–15)	< 0.001
Motivation for eating	20 (16–247)	18 (15–22)	0.139	19 (17–24)	18 (15–22)	0.044
Eating as diversion	6 (4–7)	6 (4–8)	0.489	6 (4–7)	6 (4–8)	0.628
Feeling of fullness and hunger	7 (6–9)	6 (5–8)	0.080	7 (6–9)	6 (5–8)	0.102
Bad eating habits	12 (9–14)	9 (7–12)	0.002	11 (9–13)	9 (7–12)	0.006
Meal contents	21 (18–24)	19 (16–23)	0.197	21 (18–23)	19 (16–23)	0.174
Irregular eating pattern	17 (15–20)	17 (14–20)	0.343	17 (15–19)	17 (14–20)	0.352
Total	102 (86–113)	90 (77–103)	0.009	97 (85–113)	90 (76–103)	0.007

Values are presented as median (interquartile range). Analysis was performed using the Mann–Whitney *U* test.

*MASLD* metabolic dysfunction-associated steatotic liver disease, *NAFLD* nonalcoholic fatty liver disease.

Multivariate logistic regression analyses revealed that “perception of constitution and weight” (OR 1.21; 95% CI 1.08–1.35; *P* = 0.001) and “bad eating habits” (OR 1.18; 95% CI 1.00–1.28; *P* = 0.045) were independently associated with MASLD, and only “perception of constitution and weight” was associated with NAFLD (OR 1.20; 95% CI 1.09–1.33; *P* < 0.001) (Table 3).

**Table 3:** Impact of eating behavior questionnaire category on MASLD and NAFLD.

Category	MASLD				NAFLD			
	Univariate		Multivariate		Univariate		Multivariate	
	OR (95% CI)	*P*-value	OR (95% CI)	*P*-value	OR (95% CI)	*P*-value	OR (95% CI)	*P*-value
Perception of constitution and weight	1.22 (1.11–1.34)	< 0.001	1.21 (1.08–1.35)	0.001	1.20 (1.11–1.31)	< 0.001	1.20 (1.09–1.33)	< 0.001
Motivation for eating	1.05 (0.99–1.11)	0.132	0.98 (0.89–1.09)	0.740	1.05 (1.00–1.11)	0.058	1.02 (0.93–1.11)	0.716
Eating as diversion	1.04 (0.90–1.20)	0.633	0.95 (0.76–1.18)	0.633	1.03 (0.91–1.16)	0.659	0.92 (0.77–1.11)	0.402
Feeling of fullness and hunger	1.15 (0.98–1.35)	0.090	1.01 (0.80–1.26)	0.959	1.12 (0.98–1.29)	0.100	0.99 (0.82–1.20)	0.915
Bad eating habits	1.18 (1.07–1.30)	< 0.001	1.13 (1.00–1.28)	0.045	1.13 (1.04–1.23)	0.003	1.07 (0.96–1.19)	0.208
Contents of meals	1.05 (0.97–1.30)	0.245	1.00 (0.90–1.11)	0.977	1.04 (0.98–1.11)	0.209	1.00 (0.92–1.09)	0.993
Unsteady eating pattern	1.06 (0.97–1.15)	0.219	0.96 (0.84–1.08)	0.473	1.04 (0.97–1.12)	0.246	0.96 (0.86–1.06)	0.402

Analysis was performed using the logistic regression model.

*CI* confidence interval, *MASLD* metabolic dysfunction-associated steatotic liver disease, *NAFLD* nonalcoholic fatty liver disease, *OR* odds ratio.

### Data-mining analyses for eating behavior on MASLD and NAFLD

**Figure 3:**
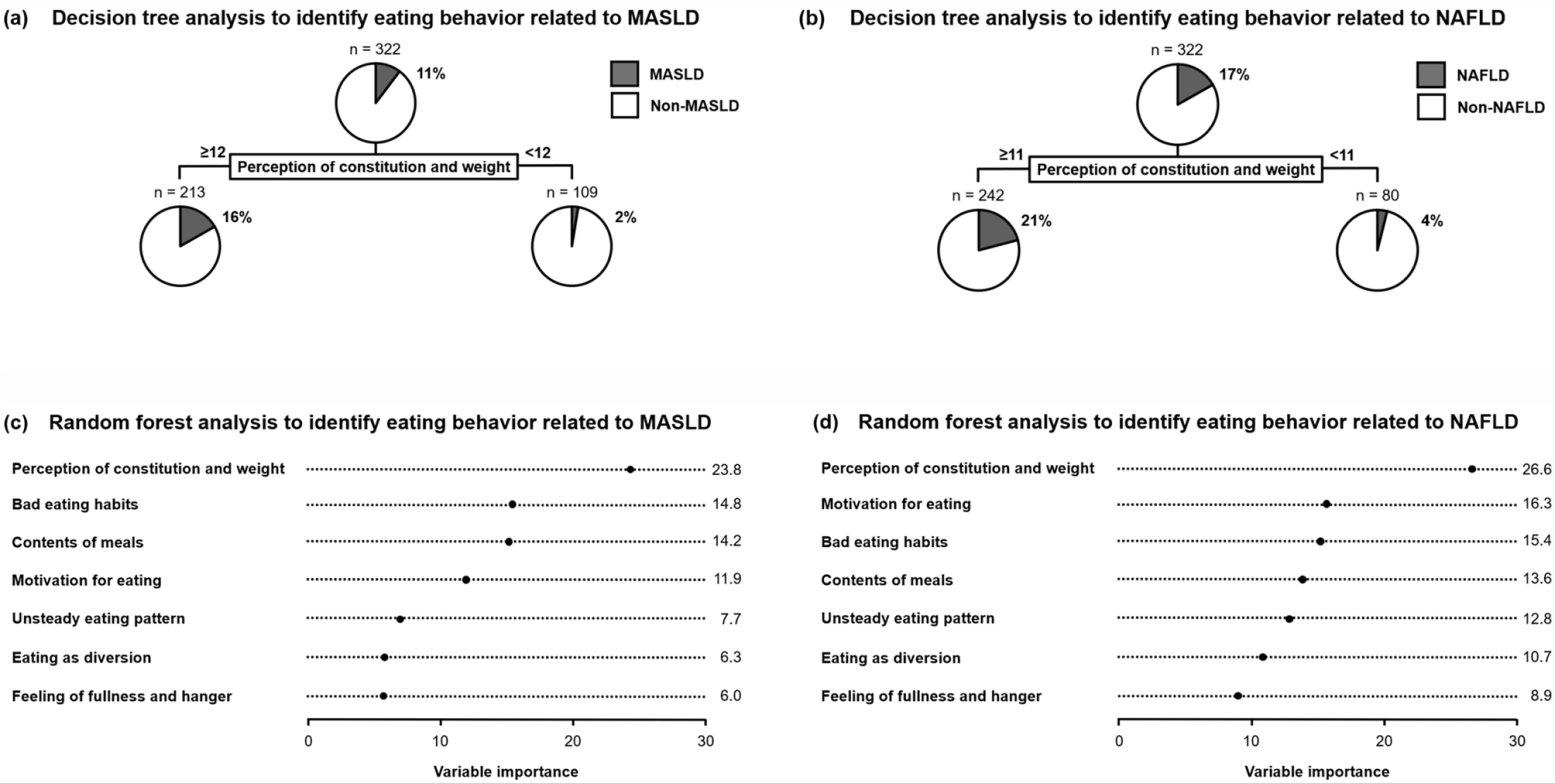
Data-mining analyses for eating behavior and MASLD and NAFLD in Japanese male young adults. The decision tree analyses revealed that “perception of constitution and weight” is the strongest classifier for (**a**) MASLD and (**b**) NAFLD. The random forest analyses revealed that “perception of constitution and weight” is the most important contributor to (**c**) MASLD and (**d**) NAFLD. *MASLD* metabolic dysfunction-associated steatotic liver disease, *NAFLD* nonalcoholic fatty liver disease.

Decision tree and random forest analyses, including all eating behavior categories, were performed to explore the association between eating behavior and MASLD and NAFLD. The decision-tree analysis revealed that the strongest classifier of MASLD and NAFLD was “perception of constitution and weight.” Participants with ≥ 12 points in “perception of constitution and weight” had a higher prevalence of MASLD than those with < 12 points (16 vs. 2%: Fig. 3a). Similarly, participants with ≥ 11 points in “perception of constitution and weight” had higher prevalence of NAFLD than those with < 11 points (21 vs. 4%: Fig. 3b). Random forest analyses demonstrated that “perception of constitution and weight” was the strongest category contributing to MASLD and NAFLD with variable importance of 23.8 and 26.6, respectively (Fig. 3c,d).

### Data-mining analyses for eating behavior questionnaire items on MASLD and NAFLD

Decision tree and random forest analyses, including all EBQ items, were performed to explore the association between questionnaire items and MASLD and NAFLD. The decision-tree analysis revealed that the strongest item associated with MASLD and NAFLD was item no. 42; “I believe I easily gain weight than others.” Participants with ≥ 2 points for item 42 had a higher prevalence of MASLD than those with < 2 points (21 vs. 4%). Similarly, participants with ≥ 2 points for item 42 had a higher prevalence of NAFLD than those with < 2 points (30 vs. 7%) (Supplementary Fig. S4). Random forest analyses also confirmed that item 42 was the strongest EBQ item contributing to MASLD and NAFLD, with a variable importance of 18.2 and 21.8, respectively (Supplementary Fig. S5).

## Discussion

The present study found that eating behavior is strongly associated with NAFLD/MASLD. Obesity can occur due to a false perception of eating habits, actual dietary consumption, and unfavorable eating habits. For instance, some patients may think that they gain body weight even by drinking water, which is a gap in the recognition of constitution and weight, and some patients may insufficiently chew food, which is a bad eating habit^14^. The EBQ was originally developed to evaluate unaware gaps and habits in eating behaviors, especially in obese patients^14^. Since behavior therapy can improve adherence to lifestyle interventions for obesity^17^, several guidelines for obesity treatment have emphasized the importance of behavior therapy in patients with obesity^13^. However, few attempts have been made to develop behavioral therapies for the treatment of NAFLD/MASLD. We hypothesized that eating behavior can affect NAFLD/MASLD and that the EBQ can be an effective modality for evaluating eating behavior among those with NAFLD/MASLD. In our study, we assessed both NAFLD and MASLD to strengthen current evidence regarding MASLD, given it is newly defined, and little relevant evidence is available to date. In addition, the association between the EBQ scores and steatotic liver disease has not been well-investigated even in the context of NAFLD.

The first intriguing finding of the present study was that eating behavior was significantly worse in participants with NAFLD/MASLD than in those without NAFLD/MASLD. The second intriguing finding of the present study was that the influence of eating behavior on MASLD was stronger than that on NAFLD. The results of the present study expand our knowledge of the pathophysiology of NAFLD/MASLD and provide an important perspective for the multidisciplinary treatment of patients with NAFLD/MASLD.

Our study showed a significant difference in eating behaviors between participants with and without NAFLD/MASLD. In previous studies, the EBQ total score was higher in obese patients than in non-obese patients and was associated with actual food consumption^18,19^. Our study revealed that young Japanese male adults with NAFLD/MASLD had significantly higher EBQ total scores than those without NAFLD/MASLD. As for eating behavior category, decision tree and random forest analyses revealed that “perception of constitution and weight” was the strongest contributor for NAFLD/MASLD. The robust association between “perception of constitution and weight” and NAFLD/MASLD can be explained by a prior study that demonstrated that “perception of constitution and weight” was strongly correlated with BMI (*r* = 0.41)^18^, which can stratify the risk of NAFLD/MASLD^20^. In fact, BMI also correlated with the EBQ total score (*r* = 0.31) and “perception of constitution and weight” (*r* = 0.47) in our study. The initial approach to behavioral therapy varies according to each patient’s problematic eating behavior; therefore, physicians should evaluate eating behavior and adopt individualized behavior therapy to improve adherence to lifestyle modifications in patients with NAFLD/MASLD^12^. Since the EBO has good internal reliability (coefficient alpha = 0.81)^18^, the novelty of our findings should be emphasized in that assessment of eating behavior using the EBQ can be a basic approach to initiating behavior therapy in young adults with NAFLD/MASLD.

The second finding of our study was that the effect of eating behavior on MASLD was stronger than that on NAFLD. Based on the subgroup analyses categorized by NAFLD and MASLD, individuals with both conditions exhibited a higher EBQ total score than those without either condition, while no statistical difference was observed between individuals with only NAFLD and those without either condition. In addition, multivariate logistic regression analysis revealed that two categories of eating behavior, “perception of constitution and weight” and “bad eating habits,” were independently associated with MASLD, whereas only one category, “perception of constitution and weight,” was associated with NAFLD. Since eating behavior can lead to obesity, our results provide a reasonable explanation for the fact that patients with metabolic dysfunction-associated fatty liver disease—the diagnosis of which includes risk factors for NAFLD—have a higher risk of obesity, fibrosis, cardiovascular disease, and mortality than those with NAFLD^21–23^. Therefore, the assessment of eating behavior can be a useful strategy to identify individuals at high risk of MASLD who require early detection and intervention, especially at a younger age.

An additional finding of our study was that we identified an effective question item to stratify the risk of NAFLD/MASLD. Participants who had ≥ 2 points in the question asking that “I believe I easily gain weight than others” had significantly higher prevalence of MASLD and NAFLD than those with < 2 points and the results were confirmed by the data-mining analysis. A recent study has shown that patients with NAFLD have good knowledge of their disease; however, approximately half of patients with NAFLD are not ready for lifestyle modifications, including diet and exercise^11^. Although participants who had ≥ 2 points in this question may have genetic or constitutional factors that can lead to obesity, our results imply that individuals with NAFLD/MASLD have unfavorable recognition of their constitution, which can limit adherence to lifestyle interventions. Thoughts can influence mood and behavior, and cognitive restructuring can be a useful method to modify an individual’s mindset to promote favorable effects on lifestyle modifications^12^. In previous studies conducted in Japan, eating behavior, assessed using the EBQ, was robustly associated with obesity and metabolic syndrome^24,25^. Therefore, recognizing eating behavior in each individual and modifying unfavorable behaviors based on the results of the EBQ can be a useful method to implement effective lifestyle modifications for NAFLD/MASLD prevention or treatment. In addition, since the EBQ has been reported to be useful in monitoring eating behavior^26^, the questionnaire is also applicable to behavior therapy and monitoring during lifestyle interventions in patients with NAFLD/MASLD.

However, some limitations of this study must be addressed. This was a single-center study that included only young Japanese male adults, and the results may not be applicable to other regions, females, and age groups. Considering the apparent sex differences in obesity, type 2 diabetes mellitus, and other metabolic abnormalities^27^, we plan to conduct a further investigation including both males and females to extend the findings of our study. Despite these limitations, our study has several strengths, including the use of a young cohort, originality, and application of data mining analysis and has meaningful implications for the association between eating behavior and NAFLD/MASLD among young male adults.

In conclusion, our study adds to the growing evidence that eating behavior is significantly worse in individuals with NAFLD/MASLD than in those without. Eating behavior of those with NAFLD/MASLD is characterized by unfavorable recognition of their constitution and weight. Furthermore, our study provides evidence that the EBQ is useful for behavioral therapy in patients with NAFLD/MASLD. Our study encourages better application of a multidisciplinary approach to maximize the benefit of lifestyle interventions in young adults and reduce the future burden of NAFLD/MASLD.

### Supplementary Information

Supplementary Information.

## Data Availability

The data analyzed in this study is availabule from the corresponding author on reasonable request.
