# Supplementary material for: Usefulness of a questionnaire for assessing the relationship between eating behavior and steatotic liver disease among Japanese male young adults
Source: Sci Rep. 2024 Jan 25;14:2194. doi: 10.1038/s41598-024-52797-8 (PMC10810865; doi:10.1038/s41598-024-52797-8)
Supplement: Supplementary file 1 — Supplementary Information. [file 41598_2024_52797_MOESM1_ESM.pdf]

Supplementary Information

Title: Usefulness of a questionnaire for assessing the relationship between eating behavior and steatotic liver disease among Japanese male young adults

Takao Miwa[1,2,3*], Satoko Tajirika[1,2,3], Tatsunori Hanai[2,3], Nanako Imamura[1], Miho Adachi[1,3], Ryo Horita[1,3], Taku Fukao[1,3], Masahito Shimizu[2,3], and Mayumi Yamamoto[1,3,4]

[1] Health Administration Center, Gifu University, Gifu, Japan

[2] Department of Gastroenterology/Internal Medicine, Graduate School of Medicine, Gifu University, Gifu, Japan

[3] Gifu University Hospital, Gifu, Japan

[4] United Graduate School of Drug Discovery and Medical Information Sciences, Gifu University, Gifu, Japan

Supplementary Table S1. Eating behavior questionnaire recommended by the Japan Society for the Study of Obesity

The following statements describe people's thoughts, feelings, and experiences. Please indicate how well each statement describes you, during the past a year, from (1) "not at all like me" (one point), (2) "sometimes like me" (two points), (3) "tendency like me" (three points), and (4) "extremely like me" (four points), by marking the correct number. Read each statement carefully and select only one answer per statement. Please do not skip any questions.

**Eating behavior questionnaire item**
1. I eat a meal fast.
2. I believe that I gain weight because I like sweets.
3. I often buy at the convenience stores.
4. I often have a midnight snack.
5. I am uncomfortable unless I keep enough food let in a refrigerator.
6. I gain weight because I lie down soon after I finish meal.
7. I have much occasions to attend dinner at drinking parties.
8. I am often told I eat a lot.
9. I get irritated when I am hungry.
10. I eat well even if I have a cold.
11. I often eat snacks.
12. I tend to eat left-over food because I don't want to waste.
13. I have space for my favorite foods even after meal.
14. I like strong seasoning.
15. I am not satisfied until my stomach is full.
16. I tend to eat when I am irritable or stressed.
17. I am not satisfied when a very few numbers of dishes are served at dinner.
18. I am a night person.
19. I like noodles.
20. I always gain weight whenever I take long holidays.
21. I often eat between meals.
22. I believe myself to gain weight even by drinking water.
23. I always store snacks around me.

24. I tend to eat when I see others eating.
25. I don't chew well.
26. I often eat out or order food delivery.
27. I don’t have a regular meal rhythm.
28. I tend to order more than I can eat at eating out.
29. I eat more Western food than Japanese food.
30. I often eat fast food such as hamburgers.
31. I can’t help but eat when I have nothing to do.
32. I regret after eating a lot.
33. I cannot help buying food more than necessary.
34. I tend to eat fruits or snacks when I see them.
35. I eat a lot at dinner compared with other meals.
36. I gain weight because my physical activity is low.
37. I have dinner late.
38. I cannot help cooling more than necessary.
39. I can't sleep when I feel hungry.
40. I often eat sweet rolls.
41. I stuff food into my mouth.
42. I believe I easily gain weight than others.
43. I like greasy food.
44. When I find something good at the grocery store, I buy it even if it is not planned.
45. I think about the next meal right after meal.
46. I often drink beer.
47. I don’t have enough time to have relax meal.
48. I skip breakfast.
49. I do not realize hungry or fullness.
50. I frequently eat food at the social opportunity.
51. I don’t eat a lot, but I can’t lose my weight.
52. I love sweets.
53. I tend not to feel hungry before meals.
54. I eat meat a lot.
55. I eat as putting food into my mouth one after another.

Supplementary Table S2. Categories of each eating behavior questionnaire item and scores

**Category**	**Questionnaire item number**	
	**Males**	**Females**
Perception of constitution and weight	2, 6, 10, 22, 36, 42, 51	2, 6, 10, 22, 36, 42
Motivation for eating	12, 13, 24, 28, 33, 34, 38, 44, 45, 50	12, 13, 17, 24, 28, 33, 38, 44, 50
Eating as diversion	5, 16, 23, 31	5, 16, 23, 31
Feeling of fullness and hunger	9, 15, 32, 53	9, 15, 32, 39, 49, 55
Bad eating habits	1, 8, 25, 41, 55	1, 8, 25, 41, 55
Contents of meals	11, 14, 26, 29, 30, 40, 43, 52, 54	3, 19, 26, 30, 40. 43, 54
Unsteady eating pattern	4, 7, 20, 21, 27, 35, 37, 47	4, 18, 20, 21, 27, 35, 37, 48

Subtotal score of each category was calculated by adding the scores of each questionnaire item.

The total score was calculated by adding the subtotal scores in all categories.

Supplementary Fig. S1. A flow diagram of the study

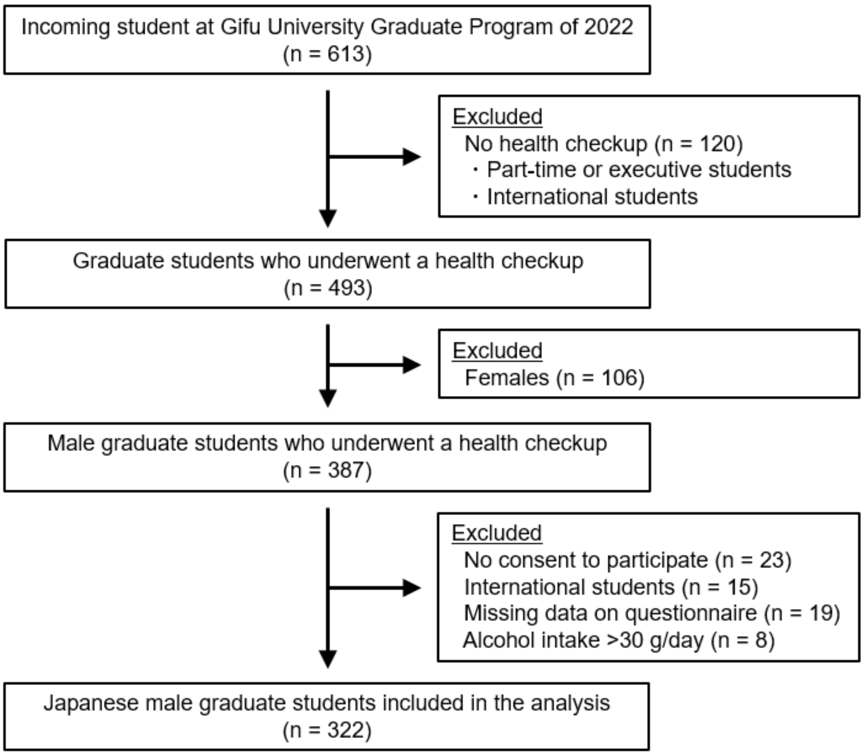

Supplementary Fig. S2. The population with NAFLD and MASLD in young Japanese male young adults

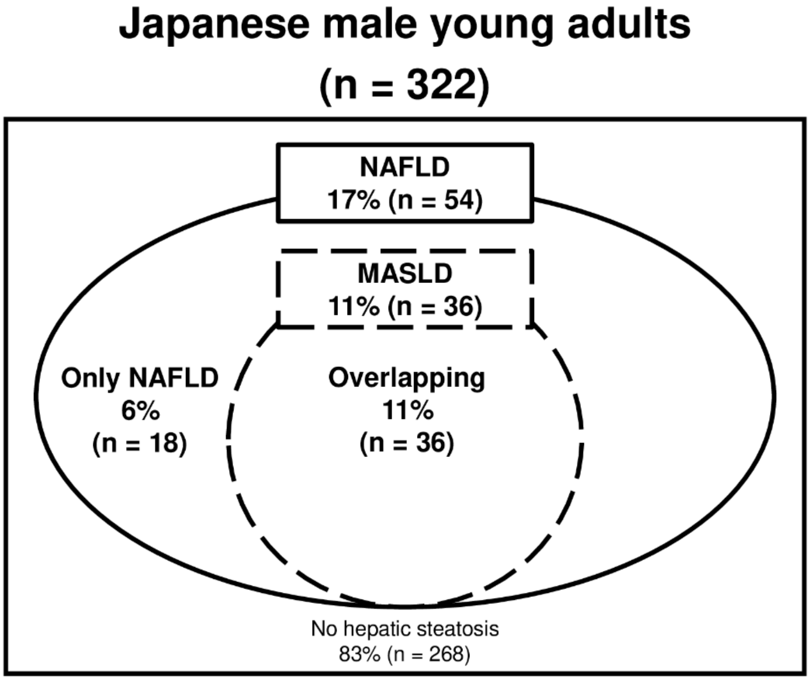

Abbreviations: MASLD, metabolic dysfunction-associated steatotic liver disease; NAFLD, nonalcoholic fatty liver disease

Supplementary Fig. S3. The association between EBQ total score and subgroups based on NAFLD and MASLD

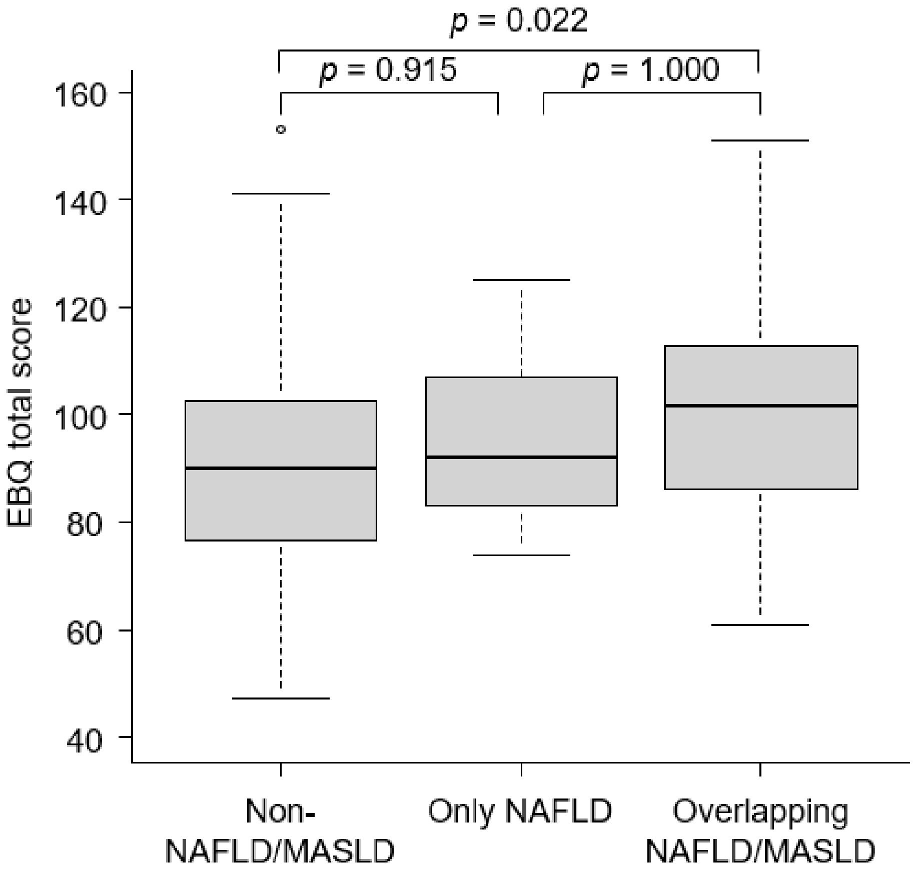

Abbreviations: EBQ, eating behavior questionnaire; MASLD, metabolic dysfunction-associated steatotic liver disease; NAFLD, nonalcoholic fatty liver disease

Supplementary Fig. S4. The decision tree analyses included eating behavior questionnaire items to identify (a) MASLD and (b) NAFLD

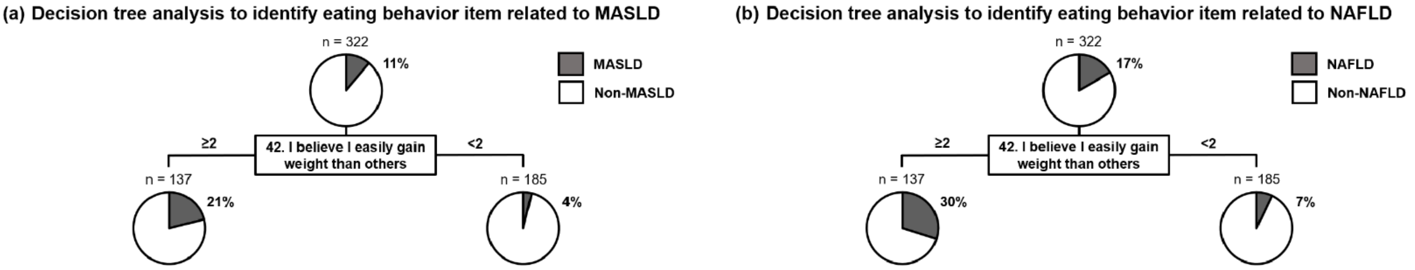

Abbreviations: MASLD, metabolic dysfunction-associated steatotic liver disease; NAFLD, nonalcoholic fatty liver disease

Supplementary Fig. S5. The random forest analyses included eating behavior questionnaire items to identify (a) MASLD and (b) NAFLD

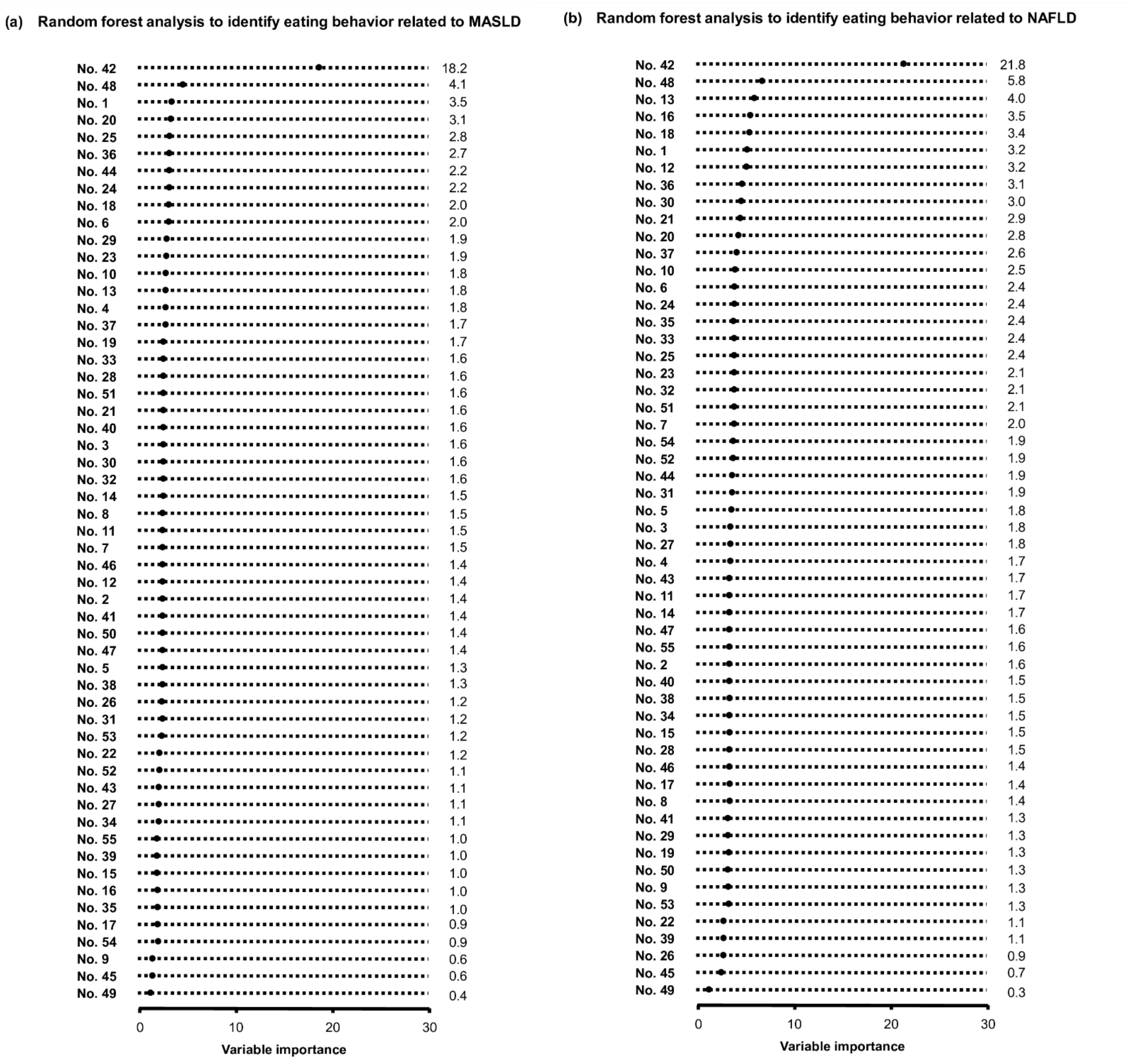

Abbreviations: MASLD, metabolic dysfunction-associated steatotic liver disease; NAFLD, nonalcoholic fatty liver disease
